# Supplementary material for: A Descriptive-Multivariate Analysis of Community Knowledge, Confidence, and Trust in COVID-19 Clinical Trials among Healthcare Workers in Uganda
Source: Vaccines (Basel). 2021 Mar 12;9(3):253. doi: 10.3390/vaccines9030253 (PMC8000597; doi:10.3390/vaccines9030253)
Supplement: Supplementary file 1 [file vaccines-09-00253-s001.zip › Supplement file 1.pdf]

Supplement file 1. Distribution of study participants with gender and regions.

| Region | District | Gender frequencies | | |
| --- | --- | --- | --- | --- |
| | | Female | Male | Total |
| Central | Entebbe | 0(0.0) | 1(1.0) | 1(1.0) |
| | Jinja | 1(1.0) | 0(0.0) | 1(1.0) |
| | Kampala | 27(26.7) | 36(35.6) | 63(62.4) |
| | Kyotera | 2(2.0) | 3(3.0) | 5(5.0) |
| | Luwero | 2(2.0) | 5(5.0) | 7(6.9) |
| | Lwengo | 0(0.0) | 1(1.0) | 1(1.0) |
| | Masaka | 0(0.0) | 1(1.0) | 1(1.0) |
| | Mityana | 0(0.0) | 4(4.0) | 4(4.0) |
| | Mubende | 0(0.0) | 1(1.0) | 1(1.0) |
| | Nakaseke | 0(0.0) | 2(2.0) | 2(2.0) |
| | Wakiso | 7(6.9) | 8(7.9) | 15(14.9) |
| | Total | 39(38.6) | 62(61.4) | 101 (100) |
| Eastern | Bukedea | 1(1.4) | 0(0.0) | 1(1.4) |
| | Bulambuli | 1(1.4) | 0(0.0) | 1(1.4) |
| | Busia | 0(0.0) | 4(5.6) | 4(5.6) |
| | Butaleja | 0(0.0) | 1(1.4) | 1(1.4) |
| | Kumi | 0(0.0) | 1(1.4) | 1(1.4) |
| | Manafwa | 0(0.0) | 1(1.4) | 1(1.4) |
| | Mbale | 4(5.6) | 2(2.8) | 6(8.3) |
| | Serere | 0(0.0) | 1(1.4) | 1(1.4) |
| | Soroti | 18(25.0) | 32(44.4) | 50(69.4) |
| | Tororo | 5(6.9) | 1(1.4) | 6(8.3) |
| | Total | 29(40.3) | 43(59.7) | 72(100) |
| Northern | Alebtong | 0(0.0) | 1(4.0) | 1(4.0) |
| | Arua | 2(8.0) | 2(8.0) | 4(16.0) |
| | Dokolo | 0(0.0) | 1(4.0) | 1(4.0) |
| | Gulu | 1(4.0) | 1(4.0) | 2(8.0) |
| | Kabong | 0(0.0) | 1(4.0) | 1(4.0) |
| | Kotido | 1(4.0) | 5(20.0) | 6(24.0) |
| | Lira | 0(0.0) | 2(8.0) | 2(8.0) |
| | Moroto | 0(0.0) | 1(4.0) | 1(4.0) |
| | Moyo | 0(0.0) | 1(4.0) | 1(4.0) |
| | Nabilatuk | 0(0.0) | 2(8.0) | 2(8.0) |
| | Napak | 0(0.0) | 1(4.0) | 1(4.0) |
| | Oyam | 0(0.0) | 1(4.0) | 1(4.0) |
| | Pader | 0(0.0) | 1(4.0) | 1(4.0) |
| | Terego | 0(0.0) | 1(4.0) | 1(4.0) |
| | Total | 4(16.0) | 21(84.0) | 25(100) |
| Western | Bushenyi | 13(21.0) | 21(33.9) | 34(54.8) |
| | Fort Portal | 0(0.0) | 2(3.2) | 2(3.2) |
| | Ibanda | 1(1.6) | 2(3.2) | 3(4.8) |
| | Isingiro | 0(0.0) | 1(1.6) | 1(1.6) |
| | Kabale | 0(0.0) | 1(1.6) | 1(1.6) |
| | Kyenjojo | 0(0.0) | 1(1.6) | 1(1.6) |
| | Lyantonde | 0(0.0) | 2(3.2) | 2(3.2) |
| | Masindi | 0(0.0) | 1(1.6) | 1(1.6) |
| | Mbarara | 2(3.2) | 11(17.7) | 13(21.0) |
| | Mitooma | 1(1.6) | 0(0.0) | 1(1.6) |
| | Ntoroko | 0(0.0) | 2(3.2) | 2(3.2) |
| | Rukungiri | 0(0.0) | 1(1.6) | 1(1.6) |
| | Total | 17(27.4) | 45(72.6) | 62(100) |

Study participants arose from 46 districts within Uganda. Generally, there were more males than females who participated in the study demonstrating gender distribution inequalities within a majority of healthcare centers in rural areas of Uganda.
