# Supplementary material for: A Descriptive-Multivariate Analysis of Community Knowledge, Confidence, and Trust in COVID-19 Clinical Trials among Healthcare Workers in Uganda
Source: Vaccines (Basel). 2021 Mar 12;9(3):253. doi: 10.3390/vaccines9030253 (PMC8000597; doi:10.3390/vaccines9030253)
Supplement: Supplementary file 1 [file vaccines-09-00253-s001.zip › Supplement file 2.pdf]

Supplementary file S2: Identifying potential challenges in COVID-19 clinical trials in Uganda

Sn	Question	Response/catogry
1.	Age	
2.	Sex (Male/Female)	
3.	Marital status: Single, Married	
4.	Education level: 1=None, 2=Certificate, 3=Diploma, 4=Bachelors, 5=Postgraduate	
5.	Occupation: 1=Clinician, 2=Nurse, 3=Pharmacist, 4=Lab personnel, 5=Support staff	
6.	District in which your health facility is located	
	Knowledge questions (correct answer 1, wrong =0 and convert scores out of 100	
7.	Beta coronaviruses include the following except: 1=MERS, 2=SARS, 3=Ebola	MERS, SARS =1 knowledge
8.	Do you know any company involved in COVID-19 vaccine/ trials development and research? 1=Yes, 2=No	Yes=1 knowledge
9.	Do you think breaking the COVID-19 circle involves vaccines and clinical trials development? 1=Yes, 2=No	Yes=1 knowledge
10.	Do you have the fear about the COVID-19 vaccine clinical trials? 1= Yes, 2=No	Yes=1 knowledge
11.	Please rank your level of fear of covid19 clinical trials: 0=No fear, 5=Extreme fear	Fear
12.	Rank the level of suspicion about the COVID-19 vaccine clinical trials? 0=No fear, 5= Extremely suspicious	Fear
13.	Willingness to participate in COVID-19 vaccine clinical trials. 0=Never, 5=Extremely ready	Fear
14.	Which is the major COVID-19 vaccines type you are comfortable with during clinical trials? 1=LAV, 2=IV, 3=HV, 4=DRV, 5=None	
15.	Willingness to participate in a rushed COVID-19 vaccine clinical trial. 0=Never, 5= Extremely ready	Fear
16.	I have been enlightened on WHO guidelines and stages for COVID-19 vaccine clinical trials	Confidence
17.	Have you ever participated in any COVID-19 vaccine clinical trial previously? 1=Yes, 2=No	Yes=1 knowlege
18.	Rank the government committed to the development of the genuine COVID-19 vaccine clinical trials? 0=Not committed, 5=Extremely committed	Confidence
19.	Confidence in the skills of Ugandans and their ability to handle the COVID-19 vaccine clinical trials: 0=Weak, 5=Extremely high	Confidence
20.	My workmates are committed to COVID-19 control guidelines and upcoming associated clinical trials?0=Weak, 5=Extremely high	Confidence
21.	There are sufficient designated medical personnel handling COVID-19 vaccine clinical trials and cases at my workplace? 0=Weak, 5=Extremely high	Confidence
22.	I have received adequate communication about the COVID-19 vaccine clinical trials in Uganda? 1=Yes, 2=No	Yes=1 knowledge
23.	My information about the planned COVID-19 vaccine clinical trials in Uganda is........	confidence

	0=weak, 5=Sufficient	
24.	Level of trust for the Uganda national regulatory guidelines for covid19 vaccine clinical trials?0=No confidence, 5= Extremely confident	Fear
25.	Access to funding in vaccine development may be a challenge for Ugandan covid19 vaccine clinical trials: 1=Not necessarily, 5=Extremely	Confidence
26.	My level of confidence in herbal and alternative medicine covid19 vaccines clinical trials being promoted in Uganda: 0=None, 5=Extremely confident	Confidence
27.	Name of Health facility/hospital	
