# Supplementary material for: A Descriptive-Multivariate Analysis of Community Knowledge, Confidence, and Trust in COVID-19 Clinical Trials among Healthcare Workers in Uganda
Source: Vaccines (Basel). 2021 Mar 12;9(3):253. doi: 10.3390/vaccines9030253 (PMC8000597; doi:10.3390/vaccines9030253)
Supplement: Supplementary file 1 [file vaccines-09-00253-s001.zip › Supplementary file 3.pdf]

Supplementary file 3: Column statistics for normality testing on knowledge, confidence and trust scores

Number of values	260	260	260
Minimum	0.0	0.2500	0.0
25% Percentile	33.33	2.000	1.600
Median	33.33	2.500	2.000
75% Percentile	50.00	3.000	3.000
Maximum	83.33	4.630	5.000
Mean	42.18	2.513	2.290
Std. Deviation	16.14	0.8161	0.9676
Std. Error of Mean	1.001	0.05061	0.06001
Lower 95% CI of mean	40.21	2.413	2.172
Upper 95% CI of mean	44.15	2.613	2.408
D'Agostino & Pearson omnibus normality test			
K2	3.707	0.2245	10.25
P value	0.1567	0.8938	0.0059
Passed normality test (alpha=0.05)?	Yes	Yes	No
P value summary	ns	ns	**
